# Clinical and Aesthetic Outcomes of Multiple Gingival Recessions Coverage with Modified Coronally Advanced Tunnel and Subepithelial Connective Tissue Graft in Maxilla and Mandible: A 2-Year Retrospective Study

**DOI:** 10.3390/ijerph191711024

**Published:** 2022-09-03

**Authors:** Izabela Skierska, Beata Wyrębek, Bartłomiej Górski

**Affiliations:** Department of Periodontology and Oral Mucosa Disease, Medical University of Warsaw, 02-097 Warsaw, Poland

**Keywords:** aesthetics, dental, gingival recession, periodontics, oral surgical procedures

## Abstract

Limited long-term data are available when analyzing gingival recession coverage between the maxillary and mandibular sites. Therefore, the aim of this study was to evaluate the influence of location (maxilla versus mandible) of multiple gingival recessions on 24 months clinical and aesthetic outcomes of modified coronally advanced tunnel with subepithelial connective tissue graft. Forty patients with multiple gingival recessions (GR) located at maxillary or mandibular teeth were treated between January 2018 and December 2019. Reduction in GR, average root coverage (ARC), complete root coverage (CRC), increase in keratinized tissue width (KTW), increase in gingival thickness (GT), and aesthetic evaluation with the root coverage esthetic score (RES) were evaluated after 24 months. Thirty patients with 270 recessions in the upper teeth and ten patients with 90 recessions in the lower teeth completed the 2-year recall. The differences between preoperative and postoperative clinical parameters showed statistical significance only within but not between groups. ARC at 2 years was 93.31% for maxillary teeth and 93.06% for mandibular teeth (*p* = 0.7906). Mean RES values were comparable for upper and lower teeth (9.25 versus 8.92, respectively, *p* = 0.6733). However, upper teeth achieved significantly higher scores for marginal tissue contour (MTC), muco-gingival junction alignment (MGJ), and gingival color (GC). Lower teeth had decreased chances of receiving better RES (OR = 0.49, CI 0.24–0.99, *p* = 0.0457) in regression analysis, when compared with upper teeth. MCAT + SCTG achieved comparably favorable 2-year outcomes for the treatment of multiple GR in upper and in lower teeth. However, the individual RES components were higher in maxillary teeth, and upper teeth had higher odds of receiving better RES.

## 1. Introduction

Gingival recession (GR), caused by migration of gingival margin apical to cementoenamel junction (CEJ), often results in aesthetic impairment, dentin hypersensitivity, and carious and non-carious cervical lesions [1]. These defects can be categorized as follows: (1) recession type 1 (RT1) when there is no loss of interproximal attachment, (2) recession type 2 (RT2) when the amount of interproximal attachment loss is lower than that of buccal attachment loss, and (3) recession type 3 (RT3) if interproximal attachment loss is greater than buccal attachment loss [2]. GR can involve one tooth (single recession-type defect) or many teeth (multiple recession-type defects). Whenever recessions are generally present, they often manifest bilaterally, both symmetrically and non-symmetrically. GR affects patients regardless of their oral hygiene level [3]. In one recent study, 91.6% of evaluated subjects were reported to have had GR (this share decreased to 70.7% when only the aesthetic aspects were analyzed) [4].

A plethora of surgical approaches can be used for the purpose of treating multiple gingival recession. The preservation of the integrity of papillae and the avoidance of vertical releasing incisions in the case of the tunnel technique, may potentially promote faster healing and positively affect clinical and aesthetic outcomes [5,6]. Such a method, originally proposed by Zabalegui et al. [7], has recently undergone a series of modifications in various studies in which full thickness flap preparation as well as application of subepithelial connective tissue graft (SCTG) were used [5,6,8,9]. In a current systematic review and meta-analysis, the utmost efficiency of the tunnel technique in treating multiple GR defects was confirmed [10]. Average root coverage (ARC) of 87.87% (±16.45) and complete root coverage (CRC) of 57.46% were reported. It has been also recently emphasized that alignment between surrogate and true end points should bear particular relevance when evaluating root coverage procedures [11]. In fact, expected aesthetic outcomes may not be achieved even in cases when CRC is provided, yet there is insufficient color match or tissue thickness. Considering the above-mentioned facts, in order to convey the outcomes more precisely, the primary focus of the root coverage procedures should be total aesthetic results.

The potential determinants of clinical outcomes resulting from application of surgical approach to multiple gingival recession can be categorized as follows: patient-dependent (general health, smoking, plaque control), pre-surgery site-specific features (recession depth and width, presence of keratinized tissue, gingival thickness, loss of interproximal attachment, tooth type, and tooth location), and surgical procedures (flap preparation technique, root surface biomodification, type of graft) [12]. It has been demonstrated that higher values of root coverage have been recorded for upper teeth [10,13,14,15]. Such discrepancies were attributed to differences in anatomical features such as: larger papillae in a maxilla, presence of lip muscles, and a minor vestibular depth in a mandible [16]. Another reason may be higher difficulty of achieving the same degrees of flap release and passive coronal positioning in case of mandibular GR.

Few studies have differentiated between the maxillary and mandibular sites, and no study investigated the RES score of root coverage with the tunnel technique and SCTG with more than 1 year of follow-up. A tendency for the relapse of the gingival margin and some deterioration of ARC and CRC over time after root coverage treatment was reported [17]. The efficacy of modified coronally advanced tunnel in the treatment of maxillary or mandibular GR has not been conclusively proved yet. Given the paucity of available results, further investigations are required. Therefore, the aim of this study was to evaluate the influence of location (maxilla versus mandible) of multiple GR RT1 and RT2 on 24-month clinical and aesthetic outcomes of the modified coronally advanced tunnel (MCAT) with SCTG.

## 2. Materials and Methods

### 2.1. Study Design and Ethical Consideration

This research was designed as a retrospective study. All surgeries were performed by the same surgeon (BG) without knowledge that the research was being developed. Only in 2022 did the authors decide to perform the present analysis as a retrospective study., Therefore, there was no bias of the operator′s clinical performance with respect to this research.

Retrospective evaluation of patients’ electronic medical records (EMR), referred to the Department of Periodontology and Oral Mucosa Diseases of the Medical University of Warsaw between January 2018 and December 2019, was carried out. Exploration of the files was performed by a specialist periodontist (BW). Charts with records of multiple gingival recessions diagnoses were included for further examination. All participants were selected among systematically healthy, non-smoking subjects with multiple gingival recessions on upper or lower incisors, canines, premolars, and molars, who were treated with MCAT and SCTG. All recessions treated between January 2018 and December 2019 in subjects who fulfilled the inclusion criteria were included in the data collection. Forty patients were ultimately included, while the total number of GRs that were analyzed amounted to three hundred sixty (270 defects in maxilla and 90 defects in mandibles).

The study protocol was approved by the Bioethics Committee (AKBE 142/2022). Informed consent was obtained from all participants, and all methods were performed in accordance with the Helsinki Declaration of 1975, as revised in Tokyo in 2013.

### 2.2. Inclusion and Exclusion Criteria

The inclusion criteria were: (1) multiple RT1 and/ or RT2 at least 1 mm deep, located at upper or lower teeth [2]; (2) full-mouth plaque score (FMPS) <20% [18]; (3) full-mouth bleeding on probing (FMBOP) <20% [19]; (4) detectable cementoenamel junction (CEJ); and (5) minimum age of 18.

The exclusion criteria were: (1) gingival recessions of type III (RT3); (2) active periodontal disease; (3) caries lesions or restorations in the cervical area; (4) systemic diseases affecting healing potential or infectious diseases; (5) use of medications affecting periodontal status; (6) smoking; and (7) pregnancy or lactation.

### 2.3. Pre-Surgical Preparations

Patients were instructed on how to use the roll technique with a soft toothbrush to eliminate harmful habits associated with gingival recessions. A professional oral hygiene procedure was also performed. Surgical intervention was scheduled only when patients could demonstrate adequate plaque control. Every patient received detailed information regarding the proposed treatment and provided informed consent.

### 2.4. Intervention

**Figure 1:**
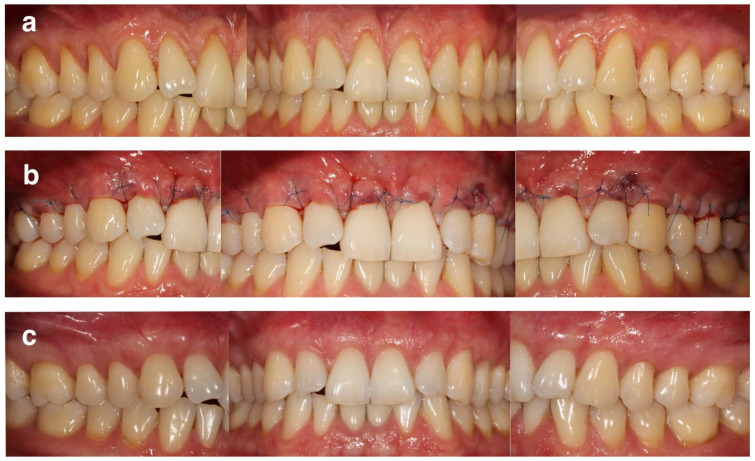
Representative case of symmetrical multiple gingival recessions at maxillary teeth (**a**) pre-operative view of gingival recessions; (**b**) immediate post-operative view; (**c**) 24 months post-operative view.

**Figure 2:**
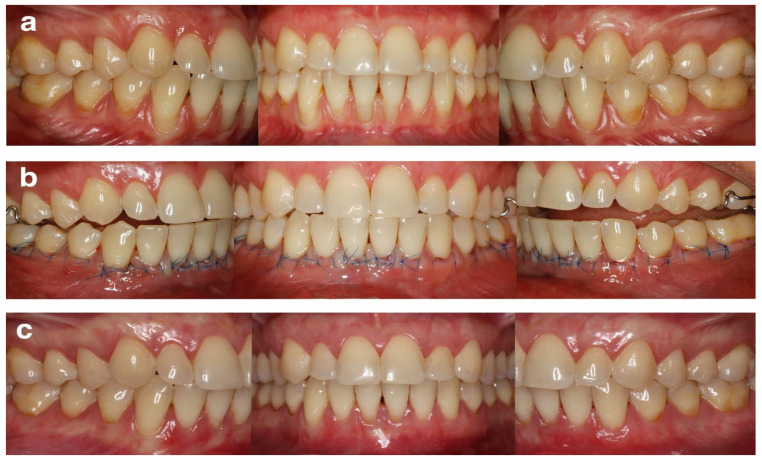
Representative case of symmetrical multiple gingival recessions at mandibular teeth (**a**) pre-operative view of gingival recessions; (**b**) immediate post-operative view; (**c**) 24 months post-operative view.

All surgical procedures were performed using the MCAT technique [20]. All recessions in maxilla or mandible were treated during the same appointment. Prior to preparing the surgical area as a full-thickness flap up to MGJ and as a split-thickness flap beyond MGJ, a local anesthesia using 4% articaine hydrochloride with adrenaline (1:100,000) (Ubistesin Forte 1.7 mL, 3-M ESPE, Saint Paul, MN, USA) was administered. The papillary regions were detached in their buccal aspects with the periosteum. Designated curettes (Gracey Curettes, Hu-Friedy, Chicago, IL, USA) were used to plane the exposed root surfaces. Thereafter, the de-epithelialized graft technique was used in order to harvest SCTG from the palate [21], whose thickness after epithelium removal was less than 1 mm. Its width was ~4 mm, while its length was in line with the length of the prepared tunnel. The area after harvesting SCTG was secured with a hemostatic sponge and cross-mattress non-resorbable sutures (Seralon 4/0 18 mm 3/8, Serag-Wiessner GmbH & Co. KG, Naila, Germany). Subsequently, SCTG was inserted into the tunnel at CEJ level and stabilized with resorbable sling sutures (PGA Resorba 6/0 11 mm 3/8, RESORBA Medical GmBH, Nürnberg, Germany). Finally, SCTG was fully covered by the coronally advanced buccal flap and secured with 6/0 non-resorbable monofilament sling sutures (Seralon 6/0 12 mm 3/8, Serag-Wiessner GmbH & Co. KG, Naila, Germany). Representative cases are shown in Figure 1 and Figure 2.

### 2.5. Post-Surgical Care and Follow-Up

As a part of the post-surgical care, first and second doses of 400 mg of ibuprofen were prescribed. They were also asked not to brush and floss the treated area for 14 days and rinse the mouth two times a day using 0.2% chlorhexidine solution. Follow-up appointments were planned to take place in 7 and 14 days and then in 1, 3, 6, 12, and 24 months. A full plaque control accompanied by hygiene instruction concerning usage of a soft toothbrush and the roll technique was conducted during each control visit. The removal of sutures took place 14 days after the surgery.

### 2.6. Clinical Measurements

Clinical parameters concerning each gingival recession were recorded and assessed before the surgery and at successive follow-up examination (24 months) under the same protocol in all instances by the same pre-assigned examiner (IS) with a graded periodontal probe (UNC probe 15 mm, Hu-Friedy, Chicago, IL, USA). The parameters being assessed were: (1) gingival recession height (GR): distance from CEJ to the gingival margin at mid-buccal point of the tooth; (2) gingival recession width (RW): horizontal distance measured between the mesial and distal margin of the recession at CEJ level; (3) probing pocket depth (PPD): distance from the gingival margin to the bottom of the gingival sulcus; (4) clinical attachment level (CAL): distance from CEJ to the bottom of the gingival sulcus; (5) keratinized tissue width (KTW): distance between the gingival margin and the muco-gingival junction (MGJ); (6) gingival thickness (GT): measured at buccal side of the tooth 3 mm apically starting from the gingival margin using endodontic file 25 ISO (Poldent, Warsaw, Poland) with a stopper positioned perpendicularly to the gingiva until root surface or the alveolar bone was reached—an electronic caliper (YATO YT-7201, Toya, Wrocław, Poland) with 0.01 mm accuracy was used to measure GT value; (7) FMPS: share of surfaces with plaque presence [18]; and (8) FMBOP: share of points that bled after gentle probing [19].

### 2.7. Aesthetic Evaluation

The aesthetic effects of the treatment were evaluated 24 months after surgery by an independent blinded examiner (MS) in line with RES [22]. The evaluation consisted of comparing photographic images before the surgery and 2 years after the procedure with a particular focus on the following variables: gingival margin (GM), soft tissue texture (STT), muco-gingival junction alignment (MGJ), marginal tissue contour (MTC) and gingival color (GC). Variables were assessed using 0–1 scoring scale, with a single exception of GM in which case a score of 0, 3, or 6 was utilized. The maximum overall score was 10.

### 2.8. Study Outcomes

A comparison of aesthetic results of MCAT + SCTG in upper teeth versus MCAT + SCTG in lower teeth after 24 months, as well as identification of parameters affecting the overall aesthetic score in each of the analyzed groups were the primary outcomes of the study. Secondary outcomes included percentages of complete root coverage and root coverage (CRC, ARC), reduction in gingival recession height (GR), gain in clinical attachment level (CAL), increase in gingival thickness (GT), and increase in keratinized tissue width (KTW).

### 2.9. Sample Size

The sample size was calculated based on an alpha error of 0.05 and a power of 0.95. For variability, data from a previous study were used as a reference [14]. The minimal clinically significant value was set at 0.5 mm. The expected effect of the difference in the reduction of recession was 0.35 between upper and lower teeth. Consequently, a minimum of 46 recessions per group was required.

### 2.10. Statistical Analysis

The data were analyzed using 3.6.1 software (R Core Team 2021). The metrics calculated were as follows: (1) recession reduction = GR0–GR12, (2) ARC = GR0– GR12/GR0 × 100%, (3) CAL gain = CAL0–CAL 12, (4) KTW gain = KTW12–KTW0, and (5) GT gain = GT12–GT0. Descriptive statistics included mean values, standard deviations (SD), frequencies, and percentages. The Shapiro–Wilk test was utilized to check for the normality of distribution of quantitative variables. As the normality was confirmed in case of all variables, it was possible to use Student’s t-test to compare means between two groups. Pearson′s chi square test was utilized to compare fractions (percentages). Statistical significance was established for *p* < 0.05.

## 3. Results

### 3.1. Patient and Defect Characteristics

Forty patients (29 females and 11 males) treated between January 2018 and December 2019 were enrolled in the study. Their mean age was 29.04 (±3.82) years (age range, 21–38), and all participants completed the 2-year recall. A total of 360 gingival recessions were treated (270 defects in the upper teeth, 90 defects in the lower teeth), with similar inter-group characteristics and tooth distribution (Table 1). No complications were observed in any participating patients during the follow-up period.

### 3.2. Clinical Outcomes

Before treatment, no statistically significant differences in the analyzed variables between evaluated groups were observed (Table 2). The change in value of those variables between starting point and measurement 24 months after the surgery was statistically significant for both groups. Similar reductions in GR and RW as well as comparable increases in KTW and GT were identified. GR height at baseline was 2.07 ± 1.04 mm for the upper teeth and 2.01 ± 1.09 mm for the lower teeth (*p* = 0.6657). The recession height at 2 years was 0.18 ± 0.60 mm (ARC 93.31%) for maxillary teeth and 0.16 ± 0.52 mm (ARC 93.06%) for mandibular teeth. CRC was achieved in 87.43% of the upper recessions and in 87.29% of the lower recessions at 24 months.

### 3.3. Aesthetic Evaluation

Two years after treatment mean RES values were comparable for upper and lower teeth (9.25 ± 1.36 versus 8.92 ± 1.40, respectively, *p* = 0.6733) (Table 3). However, the upper teeth showed significantly higher values for MTC, MGJ, and GC as compared to the lower teeth. In particular, 13.88% of the upper defects showed a MTC of zero, in contrast to 16.25% of the lower defects. Similarly, 10.22% of the upper teeth showed MGJ of zero, and 6.81% of the upper teeth showed GC of zero (for the lower defects the above-mentioned values were 17.77% and 12.22%, respectively).

Based on the results of the developed logistic binary regression model, it can be concluded that patients assessed to have a satisfactory MTC (value of 1) had an OR of 9.46 to be granted a better RES score when compared with the ones whose MTC score was zero in RES assessment (CI 4.71–19.57, *p* < 0.0001). Quite similarly, adequate STT, MGJ, and GC, were associated with the OR of 5.41 (CI 2.43–12.15, *p* < 0.0001), 5.67 (CI 1.56–12.74, *p* < 0.0001), and 5.63 (CI 2.15–14.71, *p* = 0.0003), respectively, to achieve an improved respective RES score. Recession position was also significantly associated with the final RES. Lower teeth had lower likelihoods of receiving a better RES (OR = 0.49, CI 0.24–0.99, *p* = 0.0457) when compared to upper teeth.

The multiple regression model proved that it is possible to estimate the value of RES using variables such as GM, MTC, STT, MGJ, GC, and tooth position (Table 4). All above-mentioned variables are statistically significant (*p <* 0.0001), and the *R^2^* of the developed model was 47.01%.

## 4. Discussion

There are few data in the available literature that took a comparative approach to the influence of location (maxilla versus mandible) on MCAT + SCTG used for the purpose of treating multiple GR. As far as we are aware, this study is the first that investigates the 24-month clinical and aesthetic outcomes of this surgical technique. Therefore, our results are partly comparable to those attained in other studies. A statistically significant difference between pre- and post- operative values of the analyzed variables (GR reduction, ARC, CRC, KTW gain and GT gain) was identified but only within groups (there were no statistically significant differences between groups). ARC after 2 years was 93.31% for maxillary teeth and 93.06% for mandibular teeth (*p* = 0.7906). CRC was achieved in 87.43% of the upper recessions and in 87.29% of the lower recessions. From the clinical standpoint, the results of this study do not support the hypothesis of key role of the GR location on root coverage with MCAT + SCTG. Such outcomes are consistent with the findings of other studies [3,10,13,15,23]. In a recent meta-analysis, the overall ARC of tunnel technique for multiple maxillary GR defects was 88.63 ± 7.08%, while ARC for multiple mandibular GR defects was 85.88 ± 27.77% [10]. The CRC was lower in upper GR compared to lower multiple GR defects (56.7% versus 61.35%, respectively).

Our findings are not in accordance with results from previous studies that assessed the performance of SCTG substitutes. Cieślik-Wegemund et al. [14] compared the clinical efficacy of using a tunnel technique with a collagen matrix to cover multiple recessions in the maxilla or mandible. In contradiction to our findings, the mean ARC 6 months after treatment was 96.8% in the maxilla and 81.3% in the mandible, and CRC was obtained in 2 out of 9 patients and 31 out of 39 recessions (79%) in the maxilla—0 out of 5 patients and 10 out of 20 recessions (50%) in the mandible. Quite similarly, Chaparro et al. [24] and Shepherd et al. [25] achieved greater root coverage in the maxilla than in the mandible, but they implemented acellular dermal matrix instead of SCTG. Authors concluded that the limitation of flap mobilization, the coronal advancement and direct impaired access to the operating field in the lower arch could have contributed to the lack of predictability for mandibular GR. A possible explanation might be the fact that the composition of SCTG substitutes differed substantially from autologous soft tissue grafts used in our study. According to Zucchelli et al. [21] a statistically greater increase in buccal soft tissue thickness may be achieved via de-epithelialization of the gingival graft, owing to better quality of connective tissue directly under the epithelium; this seemed to be of paramount importance since not only the amount of keratinized tissue width but also gingival thickness may serve as predictors for the stability of the gingival margin. This hypothesis is also supported by data from the present study.

According to Aroca et al. [13], the distance from the contact point to the tip of papilla (DCP) and the type of tooth significantly influenced root coverage one year after treatment of Miller class III multiple GR. The probability of achieving CRC was greater than 89% when DCP prior the surgery was less than 3 mm for teeth in a maxilla, while for teeth in a mandible, the probability was 34%. The key factor believed to be responsible for such disparities was the fact that various defect types were treated in the cited study. With that in mind, site-specific muco-gingival conditions, such as KTW and GT, should be considered when assessing the location of the tooth. In another study, the position and type of a tooth were independently associated with ARC, KTW gain, and GT gain. A logistic regression model estimated the probability of post-operative ARC > 85% and postoperative KTW > 3 mm to be significantly higher in case of premolars in comparison to incisors (OR = 1.78; 95% CI = 0.45–3.54 and OR = 0.46; 95% CI 0.27–0.75, respectively) [15]. Furthermore, in the case of mandibular teeth, the likelihood of 12-month KTW > 3 mm and GT > 2 mm was four times higher, which implied that KTW and GT gains are greater for lower teeth.

Aesthetic outcomes belong to key determinants of patient satisfaction, as they are one of the most fundamental reasons for launching such a treatment [26]. In the present study, mean RES values were comparable for upper and lower teeth (9.25 versus 8.92, respectively, *p* = 0.6733). This finding seems to suggest that the efficacy of root coverage procedures with MCAT + SCTG in maxillary GR could be similar to that achieved in mandibular GR. This seems to support the findings from previous studies [5,15,27] and is in agreement with the results of a recent systematic review and meta-analysis [28]. Nevertheless, the post-operative soft tissue quality including elements, such as gingival margin contour, color, texture, and lack of scar tissue formation, should be of equal importance to complete root coverage when assessing the results of recession treatment procedures. While CRC accounts for 60% of the RES value, the remaining part can be explained by other above-mentioned determinants, which impact smile aesthetics [2]. Consequently, the upper teeth achieved significantly higher scores for marginal tissue contour (MTC), muco-gingival junction alignment (MGJ), and gingival color (GC). The multiple regression model proved that it is possible to estimate the value of RES using variables, such as GM, MTC, STT, MGJ, GC, and tooth position. The lower teeth had smaller chances of receiving better RES (OR = 0.49, CI 0.24–0.99, *p* = 0.0457), when compared with upper teeth. As far as we are aware, there has been no study yet which compares RES values of the coverage of multiple GR in the maxilla and mandible using MCAT + SCTG. Therefore, comparing our study with other studies is difficult. In a recent study by Pietruska et al. [29], application of MCAT + SCTG resulted in total RES of 8.36. At the same time, however, other parameters, such as MGJ and GC, were higher for root coverage with MCAT + collagen matrix. Nevertheless, those findings cannot be directly compared with the results of the current research. In multivariate analysis, the greater the baseline GT, the more likely the possibility of achieving RES = 10 (OR = 5.50; 95% CI = 3.34–16.43) 12 months after the MCAT + SCTG technique [15]. Moreover, the likelihood of achieving RES = 10 increased by a factor of ten (OR = 10.23; 95% CI = 5.78–32.23) when enamel matrix derivatives were additionally applied.

Even though root coverage with SCTG is considered the gold standard, there are some clinical scenarios in which adding SCTG to MCAT may not be beneficial. According to some data, SCTG should be added beneath the flap only in sites with a thin phenotype or a reduced dimension of KTW [30,31]. The final aesthetic outcomes may even be compromised if SCTG is added in a site with an already thick gingival phenotype [32]. A recent meta-analysis showed that addition of SCTG might result in less natural tissue texture and gingival color. The authors speculated that the addition of graft in the presence of a thick gingiva at baseline, may result in a bulky and irregular gingival margin that does not follow for CEJ. Nevertheless, SCTG promoted better stability of the gingival margin and some degree of creeping attachment over time compared to other surgical approaches. Interestingly, in the present study, the logistic binary regression showed that GR with an adequate MTC (value of 1) had an OR of 9.46 of achieving a better RES score than those that received a score of zero for this parameter in RES assessment.

The current study has numerous limitations that are fully known to the authors. As the study is of retrospective nature, the risk of bias is significantly higher, which is why presented data should interpreted with caution. However, no problems related to availability of data have been encountered, as it was possible to collect them from all participants. The research was performed within the clinical environment of a university dental clinic. Identical methodology was used in every single case to achieve the expected treatment success by the same surgeon, who had no knowledge that the present research was being developed. Another plausible limitation is the disproportion between the number of evaluated maxillary GR when compared to mandibular GR, which could influence the results. However, sample size was calculated. Moreover, GR in both groups had similar baseline characteristics, which allowed effective evaluation of each surgical site. The fact that no data concerning outcomes self-reported by patients have been gathered might also be considered a drawback of the study. Further randomized clinical trials are required for assessing our results in terms of recurrence of GR. Multicenter studies may favor the inclusion and evaluation of larger samples of patients and consequently the achievement of statistical power.

## 5. Conclusions

The study showed that the location of the recession (maxillary versus mandibular) did not affect the clinical results of recession coverage with MCAT + SCTG. The technique achieved comparably favorable 2-year results in the treatment of multiple GRs in both upper and lower teeth. However, the location of the recession may influence the aesthetic results of such a treatment. Considering the individual components of the aesthetic scale (such as soft tissue color, marginal tissue contour, and alignment of the mucogingival junction), higher values were achieved after treatment of recessions in the maxilla.

## Figures and Tables

**Table 1:** Characteristics for the study groups.

Variables	Upper Teeth (*N* = 30; *n* = 270)	Lower Teeth (*N* = 10; *n* = 90)	*p*
Sex (*n*)			
Women	21	8	0.5396
Men	9	2	
Age (mean, SD)	29.58 (3.81)	28.50 (3.72)	0.6157
Tooth type (*n*)			
Incisors	72	22	0.6112
Canines	58	18	
Premolars	100	40	
Molars	40	10	
Type of GR according to Cairo (*n*, %)			
RT1	148 (54.81%)	47 (52.22%)	0.5551
RT2	122 (45.19%)	43 (47.78%)	

*N* number of patients, *n* number of defects, SD standard deviation, GR gingival recession, RT recession type.

**Table 2:** Clinical parameters (mean and standard deviation) at baseline and 24 months after surgery.

	Baseline	24 Months	*p*
GR upper teeth (mm)	2.07 ± 1.04	0.18 ± 0.60	<0.0001 *
GR lower teeth	2.01 ± 1.09	0.16 ± 0.52	<0.0001 *
*p*	0.4143	0.6657	
ARC upper teeth (%)	-	93.31 ± 21.82	-
ARC lower teeth	-	93.06 ± 22.49	-
*p*		0.7906	
CRC upper teeth (%)	-	87.43	-
CRC lower teeth	-	87.29	-
*p*		0.5798	
GR red upper teeth (mm)	-	1.89 ± 1.03	-
GR lower teeth	-	1.84 ± 1.13	-
*p*		0.9554	
RW upper teeth (mm)	3.51 ± 1.51	0.37 ± 1.11	<0.0001
RW lower teeth	3.51 ± 1.49	0.43 ± 1.28	<0.0001
*p*	0.9981	0.1038	
PPD upper teeth (mm)	1.43 ± 0.54	1.41 ± 0.57	0.8239
PPD lower teeth	1.46 ± 0.54	1.45 ± 0.64	0.8586
*p*	0.4211	0.5635	
CAL upper teeth (mm)	3.44 ± 1.15	1.21 ± 0.95	<0.0001 *
CAL lower teeth	3.29 ± 1.20	1.19 ± 0.95	<0.0001 *
*p*	0.5671	0.7627	
KTW SCTG + EDTA (mm)	2.67 ± 1.33	3.49 ± 1.29	<0.0001 *
KTW SCTG	2.58 ± 1.27	3.39 ± 1.30	<0.0001 *
*p*	0.3029	0.29105	
KTW gain upper teeth (mm)	-	0.74 ± 1.17	-
KTW lower teeth	-	0.81 ± 0.94	-
*p*		0.3949	
GT upper teeth (mm)	1.41 ± 0.59	2.25 ± 0.78	<0.0001 *
GT lower teeth	1.43 ± 0.60	2.26 ± 0.72	<0.0001 *
*p*	0.8718	0.9001	
GT gain upper teeth (mm)	-	0.80 ± 0.72	-
GT gain lower teeth	-	0.84 ± 0.62	-
*p*		0.7817	

GR gingival recession height, ARC average root coverage, CRC complete root coverage, GR red gingival recession reduction, RW gingival recession width, PPD probing pocket depth, CAL clinical attachment level, KTW keratinized tissue width, GT gingival thickness, * statistically significant (*p* ≤ 0.05).

**Table 3:** Evaluation of aesthetic outcomes after 12 months (mean and standard deviation).

	GM	MTC	STT	MGJ	GC	RES
Upper teeth	5.66 ± 0.96	0.88 ± 0.33	0.89 ± 0.31	0.93 ± 0.26	0.95 ± 0.22	9.25 ± 1.36
Lower teeth	5.58 ± 1.18	0.77 ± 0.42	0.85 ± 0.35	0.83 ± 0.37	0.89 ± 0.32	8.92 ± 1.40
*p*	0.7243	0.0006 *	0.1075	<0.0001 *	<0.0001 *	0.6733

GM gingival margin, MTC marginal tissue contour, STT soft tissue texture, MGJ muco-gingival junction alignment, GC gingival color, RES root coverage esthetic score, * statistically significant (*p* ≤ 0.05).

**Table 4:** Multiple linear regression model identifing variables that determine *R*^2^ the final RES. The model was statistically significant with a *R*^2^ adjusted to 0.4701 and *p* < 0.0001.

Variable.	Coefficient	Standard Error	*p*	95% Confidence Interval
Gingival margin (GM)	5.6297	0.5896	<0.0001 *	4.47–6.79
Marginal tissue contour (MTC)	1.4107	0.1433	<0.0001 *	1.13–1.69
Soft tissue texture (STT)	0.9465	0.1653	<0.0001 *	0.62–1.27
Muco-gingival junction alignment (MGJ)	0.7957	0.1671	<0.0001 *	0.47–1.12
Gingival color (GC)	1.0925	0.2012	<0.0001 *	0.70–1.49
Recession location (upper versus lower)	−0.3931	0.1252	<0.0001 *	−0.64–−0.15
Constant	0.3097	0.6758	0.64708	−1.02–1.64

* statistically significant (*p* ≤ 0.05).

## Data Availability

Not applicable.
